# RETRACTED: Ashar et al. Palmitic Acid Impedes Extravillous Trophoblast Activity by Increasing MRP1 Expression and Function. *Biomolecules* 2022, *12*, 1162

**DOI:** 10.3390/biom14040453

**Published:** 2024-04-08

**Authors:** Yunali Ashar, Qiuxu Teng, John N. D. Wurpel, Zhe-Sheng Chen, Sandra E. Reznik

**Affiliations:** 1 Department of Pharmaceutical Sciences, St. John’s University, Queens, NY 11439, USA; 2 Departments of Pathology and Obstetrics and Gynecology and Women’s Health, Albert Einstein College of Medicine, Bronx, NY 10461, USA

The *Biomolecules* Editorial Office retracts the article, “Palmitic Acid Impedes Extravillous Trophoblast Activity by Increasing MRP1 Expression and Function” [1], cited above.

Following publication, concerns were brought to the attention of the publisher regarding a number of partial figure duplications within this article [1].

Adhering to our complaints procedure, an investigation was conducted by the Editorial Office and Editorial Board that confirmed the partial overlap of a number of panels and Western blots contained with Figures 3–6 and 8, representing different experimental conditions. Given the extent and impact of these duplications on the validity of the overall findings, the Editorial Office, Editorial Board, and the authors have decided to retract this article [1] as per MDPI’s retraction policy (https://www.mdpi.com/ethics#_bookmark30, accessed on 29 March 2024) and in line with the Committee on Publication Ethics retraction guidelines (https://publicationethics.org/retraction-guidelines, accessed on 29 March 2024).

This retraction was approved by the Editor-in-Chief of the journal *Biomolecules*.

The authors agreed to this retraction.
